# The Mediterranean Habitat of the Nile Soft-Shelled Turtle (*Trionyx triunguis*): Genomic and Reproductive Insights into an Endangered Population

**DOI:** 10.3390/ijms26188822

**Published:** 2025-09-10

**Authors:** Adi Gaspar, Larissa S. Arantes, Talya Ohana, Yair E. Bodenheimer, Gili Tikochinski, Opal Levy, Bar J. Mor, Muriel Vainberg, Tomer Gat, Susan Mbedi, Sarah Sparmann, Oğuz Türkozan, Yaniv Levy, Noam Leader, Dana Milstein, Camila J. Mazzoni, Yaron Tikochinski

**Affiliations:** 1 Ruppin Academic Center, Faculty of Marine Sciences, Emek Hefer 4025000, Israel; gaspar.adi@gmail.com (A.G.); talya5994@gmail.com (T.O.); yairbod2@gmail.com (Y.E.B.); gilitiko@gmail.com (G.T.); opalevy199@gmail.com (O.L.); barmor1211@gmail.com (B.J.M.); murielvainberg0@gmail.com (M.V.); gatomer.98@gmail.com (T.G.); 2 Department of Genetics, Ecology and Evolution, Leibniz Institute for Zoo and Wildlife Research (IZW), Forschungsverbund Berlin e.V., 10315 Berlin, Germany; larissarantes1@hotmail.com (L.S.A.); mbedi@izw-berlin.de (S.M.); sasparmann@zedat.fu-berlin.de (S.S.); mazzoni@izw-berlin.de (C.J.M.); 3 Department of Biology, Faculty of Science, Aydın Adnan Menderes University, 09010 Aydın, Türkiye; oguz.turkozan@gmail.com; 4 Israeli Sea Turtle Rescue Center, Israel Nature and Parks Authority, Michmoret 4029700, Israel; yaniv@npa.org.il; 5 Science and Conservation Division, Israel Nature and Parks Authority, Jerusalem 9411702, Israel; leader@npa.org.il (N.L.); danam@npa.org.il (D.M.)

**Keywords:** conservation genomics, mtDNA, D-loop, heteroplasmy, 3RADseq, kinship analysis, multiple paternity, sperm storage

## Abstract

The Mediterranean soft-shell turtle (*Trionyx triunguis*) is classified as critically endangered by the IUCN. Effective conservation requires a clear understanding of its reproductive strategies and population structure. By combining mitochondrial DNA tandem repeat-region profiling with genome-wide SNP data obtained through 3RADseq, we gained high-resolution insights into the genetic composition and breeding behavior of Mediterranean populations. Our results revealed complex reproductive dynamics, including multiple paternity, sperm storage, and repeated nesting within a single season—strategies that enhance genetic diversity in small, fragmented populations. Using SNP-based kinship inference, we estimated the number of breeding females and identified full and half-sibling groups, offering a robust genomic framework for assessing population size and structure. Genetic similarity patterns highlighted moderate differentiation among Israeli river populations, suggesting some connectivity, while samples from Türkiye were clearly distinct, reflecting long-term geographic and genetic separation. This integrative approach provides a scalable, repeatable tool for long-term monitoring. The combined use of maternal and biparental markers enables detailed tracking of genetic diversity, breeding contributions, and demographic trends—key elements for designing informed, adaptive conservation strategies.

## 1. Introduction

The Nile soft-shelled turtle (*Trionyx triunguis*), is the only extant species of its genus, as first suggested by Meylan [1]. It is a large aquatic reptile belonging to the family Trionychidae within the suborder Cryptodira. Members of Trionychidae are among the most morphologically specialized turtles, characterized by a flattened, leathery carapace without scales, elongated snorkel-like snouts, and highly mobile necks, adaptations for a fully aquatic lifestyle [1,2]. Comprising 13 genera and over 30 species, this family is distributed across all continents except Antarctica and includes some of the largest and most threatened freshwater turtles [3].

Phylogenetic analyses combining mitochondrial and nuclear markers with morphological data place *T. triunguis* as a sister lineage to the giant soft-shells Pelochelys and Chitra, though this relationship is supported primarily by Bayesian inference and remains distant [3,4]. First described by Forsskål in 1775, the species remains relatively understudied despite its broad Afro-Mediterranean distribution.

*T. triunguis* inhabits both freshwater and brackish environments, including rivers, lakes, estuaries, and coastal marine habitats [5,6]. Its geographic range spans major freshwater systems of sub-Saharan and eastern Africa, including the Nile River and its tributaries in Sudan, Kenya, and Uganda; lakes Turkana and Albert; and river systems in Ethiopia, Somalia, and Tanzania. In western and central Africa, *T. triunguis* occurs from the Cunene River in Angola through Gabon, the Congo Basin, Nigeria, Ghana, and as far west as Senegal and Mauritania [7,8]. Mediterranean populations are limited to coastal streams and estuaries in Türkiye, Syria, Lebanon, and Israel, and are geographically isolated from African populations. Molecular studies have confirmed this genetic separation [4,9].

Early observations by Stanley [5] proposed that the species’ tolerance for brackish and marine waters may have facilitated dispersal into Mediterranean coastal streams via estuarine corridors or flood events. Today, marine occurrence is well documented, especially during winter months, though this behavior also increases exposure to threats such as fisheries bycatch [10,11].

Despite its broad distribution, *T. triunguis* faces severe conservation challenges. The Mediterranean subpopulation is listed as Critically Endangered by the IUCN (CR C2a), with fewer than 1000 estimated mature individuals [10]. Major threats include habitat degradation, nest predation, sand mining, pollution, bycatch, and disturbance from human activity. In Türkiye, the Dalyan and Dalaman populations are considered among the last strongholds, although even these face declining reproductive success due to tourism and boating pressure [12,13]. Trawl surveys off the Turkish coast have recorded over 400 individuals captured in a single season, underscoring both their use of marine habitats and vulnerability to industrial fishing [11].

In Israel, the Alexander River once supported a robust *T. triunguis* population, but a major flood in 1992 severely reduced its numbers to around 50 individuals [14]. Other Israeli populations in the Taninim, Naaman, Kishon, and Yarkon rivers remain small and are likely stagnant. A population in the Hula Nature Reserve was artificially established in the 1960s through the introduction of captured turtles. In recent years, after it became clear that this population negatively impacted native species such as nesting birds, the Israel Nature and Parks Authority began relocating individuals to the Yarkon River, primarily by transferring hatchlings from nests found in the reserve.

Over the past two decades, molecular studies have focused on understanding the genetic diversity and structure of *T. triunguis*, primarily through mitochondrial DNA (mtDNA) markers. The mitochondrial control region (D-loop) is a highly variable, non-coding segment often used in population genetics, phylogeography, and conservation studies due to its maternal inheritance, high mutation rate, and lack of selective constraints.

Güçlü et al. [9] analyzed mtDNA from 22 Mediterranean and four African individuals, identifying four unique haplotypes in the African group, while all Mediterranean samples shared a single haplotype—suggesting recent colonization, possible marine gene flow, and historical female nest-site fidelity. A follow-up study by Güçlü et al. [4], which examined 102 individuals using both mtDNA (D-loop) and nine microsatellite loci, confirmed significantly higher genetic diversity in Africa than in Mediterranean populations, likely reflecting bottlenecks or founder effects. Gidiş et al. [15] supported the existence of two major conservation units—continental Africa and the Mediterranean Basin—based on both mtDNA and nuclear markers. Other studies have attempted to improve the resolution of population structure by incorporating additional markers. Shanas et al. [16] used amplified fragment length polymorphism (AFLP) and mtDNA cytochrome b to compare Israeli and Turkish populations, finding no clear differentiation, but emphasizing the need for higher-resolution genomic tools. Addressing this, Güçlü et al. [17] isolated 13 polymorphic microsatellite loci from Turkish samples. These were later used by Güçlü & Bozdoğan [18] to study five Turkish populations, revealing very low nuclear diversity—especially in comparison to marine turtles such as *Chelonia mydas* and Caretta caretta—and signs of past inbreeding and bottlenecks. Collectively, these studies underscore the need for more robust nuclear and mitochondrial datasets to fully understand the population structure and conservation needs of *T. triunguis*.

In reptiles—and particularly sea turtles—the 3′ end of the mitochondrial D-loop often contains tandem repeats that vary in number and sequence, making them useful markers for individual and population-level identification [19,20]. In green turtles (*Chelonia mydas*), such repeats help define rookeries and management units [21,22]. In *T. triunguis*, the D-loop contains a tandemly repeated 8-bp motif (“TATATATC”) at its 3′ end, which exhibits high variability in repeat number. This region has proven valuable for assessing haplotype diversity and identifying heteroplasmic patterns that further resolve maternal lineages [23]. Heteroplasmy provides an intra-individual source of genetic diversity that may shift toward fixation or loss across generations, thereby influencing haplotype stability and shaping population-level evolutionary patterns [23]. In this study, we combine gel electrophoresis-based size profiling and sequence analysis of this repeat region to assist in maternal identification.

To complement mitochondrial data, we also applied double-digest restriction site-associated DNA sequencing (3RADseq), a powerful technique that enables genome-wide discovery of single-nucleotide polymorphisms (SNPs) without requiring prior genomic information. By sequencing DNA flanking restriction enzyme cut sites, 3RADseq generates thousands of polymorphic markers across both coding and non-coding regions, making it especially useful for non-model species such as *T. triunguis* [24,25]. This reduced-representation approach is cost-effective, scalable, and well-suited for studies of population structure, gene flow, and genetic diversity. In this study, 3RADseq was used to generate a high-resolution genomic dataset for *T. triunguis* populations in Israel and Türkiye.

The objectives of this work address critical conservation concerns associated with small and isolated populations, which are particularly vulnerable to reduced genetic variability resulting from genetic drift and inbreeding depression. To help mitigate these risks, this study aims to establish a foundation for improved in situ genetic monitoring of the wild *T. triunguis* population in the Mediterranean region, beginning with the population in Alexander River.

Specifically, we aim to evaluate the extent of genetic variability within the Alexander River population and compare it with other known populations in Israel and Türkiye. By combining mitochondrial haplotyping with genome-wide nuclear markers, we also seek to gain novel insights into key reproductive behaviors, including nest site fidelity (philopatry), multiple paternity, and the capacity of females to lay multiple clutches within a breeding season. In addition, we assess the degree of genetic connectivity between the Alexander and nearby Taninim River populations.

Together, these objectives aim to enhance our understanding of the genetic structure, reproductive dynamics, and conservation needs of *T. triunguis*, and to inform the development of effective, science-based management strategies for this endangered species.

## 2. Results

### 2.1. mtDNA Analysis

**Figure 1:**
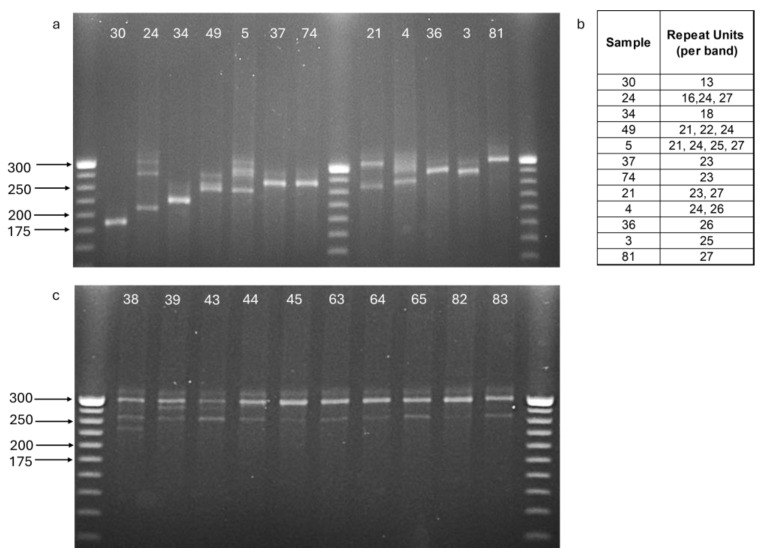
PCR amplification of the mitochondrial tandem repeat region separated on a 3% agarose gel and visualized using GelRed staining. A 25 bp DNA ladder was loaded for size calibration. (**a**) 12 samples (IDs above each lane) demonstrating different patterns of the mitochondrial tandem repeat region. (**b**) Repeat unit counts are indicated in the table. (**c**) 10 offspring (IDs above each lane) of the same female, demonstrating heteroplasmic patterns among siblings.

A detailed list of the 139 samples used in this study is provided in Table S1. Mitochondrial DNA (mtDNA) haplotypes were defined using a combination of gel electrophoresis and sequencing. Comparable samples were run in adjacent wells to detect size differences or confirm similarities in the amplified repeat region. In total, more than 40 distinct haplotypes were identified, providing valuable resolution for maternal lineage assignment. Figure 1a illustrates 11 distinct haplotypic patterns observed among 12 samples, from 11 unrelated females. The number of repeat units of 8 bp corresponding to each band in these haplotypes is provided. In contrast, Figure 1b displays 10 offspring of the same female, some of which are half-siblings (sired by different males). Subtle variations in heteroplasmic patterns among siblings highlight the high mutability and discriminatory power of this polymorphic mitochondrial region. As shown in Figure 1b, siblings inherit differing proportions of mtDNA molecules that vary in repeat number, due to a mitochondrial bottleneck effect—whereby only a small, random subset of the mother’s heteroplasmic mtDNA pool is transmitted to each offspring. While all individuals share a dominant band of 27 repeats, minor bands appear in different proportions, reflecting this stochastic sampling process. No clear correlation was observed between haplotype structure, repeat number, or heteroplasmic pattern, and population or geographic origin.

### 2.2. RADseq Analysis

The 3RAD data consisted of an average of 4,497,777 raw reads per individual, 61.8% of which remained after preprocessing (Table S2). Across all samples of DNA which have been successfully sequenced (*n* = 140, one sample sequenced twice for control), 3RAD data was generated in two separate runs of STACKS, for 89, 8, 34 and 8 individuals from sampling locations, Alexander, Taninim, Hula and Türkiye, respectively. After performing the STACKS reference-based pipeline, the average per sample locus coverage was 65.1 ± 16.2x and 94.1 ± 14.7x for All (*n* = 140) and only Hula (*n* = 34), respectively. After additional filtering steps with VCFtools, two VCFs were obtained, containing 6115 and 3565 novel SNPs that were used for the genetic analysis of *T. triunguis* at different sampling sites.

### 2.3. Population Structure

To evaluate population genetic structure, we analyzed SNP data from four sampling locations: Alexander, Taninim, Hula, and Türkiye. The Alexander River population, represented by the largest sample set, was the primary focus of this analysis. The other populations, represented by fewer individuals, served as reference points for comparison. Pairwise Fst values revealed moderate to high levels of genetic differentiation between populations. The Taninim population was genetically closest to Alexander (Fst = 0.084), while Türkiye (Fst = 0.170) and Hula (Fst = 0.187) showed greater divergence. From the Taninim perspective, higher differentiation was again observed with Türkiye (Fst = 0.144) and Hula (Fst = 0.242). The greatest genetic divergence was recorded between Hula and Türkiye (Fst = 0.302), suggesting strong historical or demographic separation. To complement these findings, we calculated ΦPT values, which account for both allele frequencies and genotype distribution. ΦPT values followed the same trends as Fst but were consistently higher, indicating more pronounced genotypic divergence (Table 1). An Analysis of Molecular Variance (AMOVA) revealed that most genetic variation occurred within individuals (68%), with 14% attributed to variation among individuals within populations and 18% among populations. All comparisons were statistically significant (*p* < 0.001), supporting the existence of distinct genetic structuring across the sampled regions.

**Figure 2:**
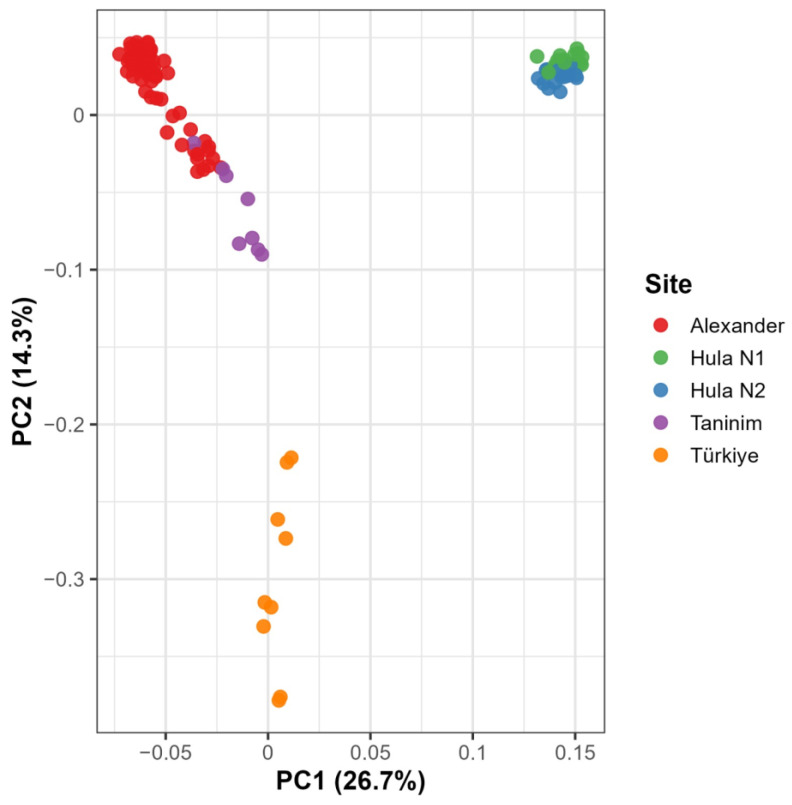
Principal components analysis (PCA) based on SNP data from 139 *T. triunguis* individuals across four sampling locations (Alexander, Taninim, Hula, and Türkiye). One Turkish individual was analyzed twice, shown as two overlapping points at the bottom. The analysis was performed using a variance-standardized relationship matrix. PC1 and PC2 explain 41% of the total genetic variation. The Hula hatchlings are divided into two nests.

Figure 2 illustrates the genetic clustering of individuals from four geographic populations based on Principal Component Analysis (PCA) of 140 samples. The first two principal components together explain 41% of the total genetic variance. Samples from the Hula population, though divided into two nests, form a tight and distinct cluster, clearly separated from all other groups. In contrast, samples from the Alexander and Taninim Rivers form partially overlapping but distinguishable clusters. The Türkiye population forms a well-separated cluster, distinct from the Israeli populations, with internal variation along PC2 that appears to reflect differences among the Turkish sampling sites.

### 2.4. Normalized-IBS (Identity by State)

Figure S1 presents a heatmap of Normalized-IBS (Identity by State) values among all individuals, capturing varying degrees of genetic relatedness and potential kinship. Higher values indicate closer genetic similarity, while lower or negative values reflect greater divergence. Table 2 summarizes the average pairwise Normalized-IBS values between the major sampling locations. The highest genetic similarity was observed between the Alexander and Taninim populations (0.12), suggesting moderate genetic connectivity. The Hula population showed low similarity to Alexander (0.06) and Taninim (0.04), while the Türkiye population exhibited the lowest or even slightly negative values when compared to all Israeli populations, indicating a high degree of genetic differentiation.

Full siblings are identifiable in the heatmap by elevated Normalized-IBS values. Their shared maternity was confirmed by nesting data and identical D-loop haplotype profiles. For instance, sibling pairs such as T37–T74 and T38–T81, which originated from different nests, display high IBS values indicative of full sibling relationships (Figure S1). The average Normalized-IBS for full siblings varied slightly among populations, likely reflecting differences in parental relatedness and statistical variance in allele sharing. The high average values among Turkish full siblings (0.64) are probably a result of the small sample number (8), compared to the Israeli population, with values of 0.56, 0.55 and 0.52 for Alexander, Hula and Taninim, respectively, as calculated from Figure S1.

### 2.5. The Alexander Population

**Figure 3:**
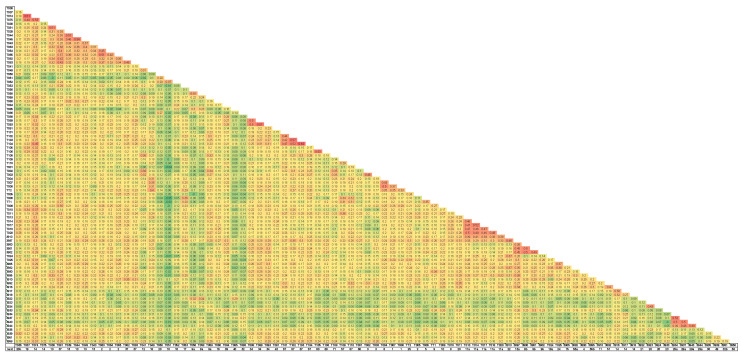
Heatmap of Normalized Identity-by-State (IBS) values for 87 *T. triunguis* individuals sampled from the Alexander River population. The color gradient represents pairwise genetic similarity, with red indicating high genetic identity and green indicating low identity. Nest designations are shown below the sample numbers.

To assess kinship patterns within the Alexander River population, we generated a heatmap of Normalized Identity-by-State (IBS) values for 87 individuals (Figure 3). These normalized values, cross-referenced with maternal lineage assignments based on mtDNA haplotyping, facilitated the identification of full and half siblings originating from the same female. The average Normalized-IBS value for full siblings was 0.49 ± 0.05, based on 46 full-sibling comparisons. For half siblings, the average was 0.30 ± 0.05, based on 13 pairwise values (see Table S3). The relatively high mean and standard deviation among half siblings likely reflect some degree of inbreeding within the population.

These data enabled further identification of potential half-siblings and the detection of repeated mating events involving specific females and males. In total, we estimate that 44 females and 36 males participated in reproduction during the five-year period between 2017 and 2021, with an additional mating pair identified in a nest from 2011 (see Table S1). Evidence of multiple clutches laid by the same female within a single year was documented (e.g., nests 2, 13 and 37). Some females demonstrated the ability to store sperm and produce full-sibling clutches over multiple nesting events (e.g., nests 14 and 19). Multiple paternity was also observed in several nests (e.g., nests 9 and 57).

### 2.6. The Two Hula Nests

**Figure 4:**
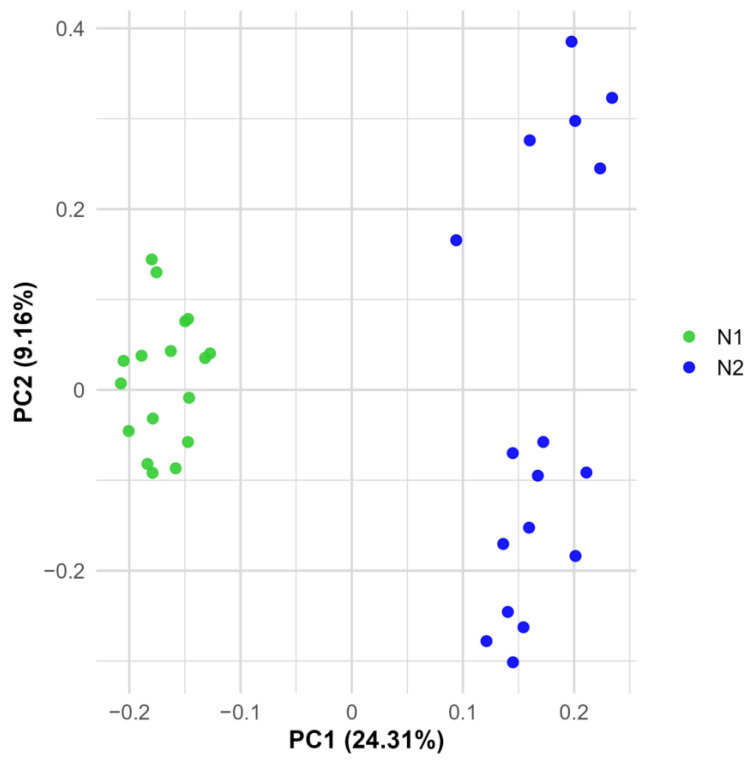
Principal components analysis (PCA) based on SNP data from 34 *T. triunguis* individuals from two nests (N1 and N2) sampled in Hula Nature reserve in Israel.

To investigate fine-scale genetic structure among closely related individuals, the VCF file of the 34 Hula samples, originating from two nests, was analyzed separately. Principal Component Analysis (PCA) revealed clear patterns of genetic differentiation. The first two principal components together explained 33.46% of the total genetic variation within this subset (n = 34). As shown in Figure 4, PC1 clearly distinguishes the two nests into separate genetic clusters. Interestingly, PC2 further separates Nest 2 into two distinct subgroups: subgroup 2a (T115, T122, T124, T125, T132, T134) and subgroup 2b (T118, T123, T126, T127, T128, T129, T135, T136, T137, T139, T142).

This pattern was further reflected in the Normalized-IBS heatmap (Figure S2). The average pairwise Normalized-IBS between individuals from different nests was 0. Within Nest 1, the average was 0.49, while within Nest 2 it was 0. When Nest 2 was split into the two subgroups, the within-group averages were 0.50 for subgroup 2a and 0.43 for subgroup 2b, with an average of 0.32 between the two subgroups. This data suggests the occurrence of multiple paternity.

## 3. Discussion

The Nile soft-shelled turtle (*T. triunguis*) represents a relic lineage within Trionychidae, with adaptations for aquatic life and a historically broad Afro-Mediterranean distribution. Yet, its Mediterranean subpopulations are highly fragmented and critically endangered [9,10]. Genomic data from these small populations is vital for informed conservation, particularly for evaluating genetic health, reproductive behavior, and lineage persistence. In this study, we combined mitochondrial tandem repeat-region profiling with 3RADseq-derived SNP analysis to provide the most comprehensive genomic assessment to date of *T. triunguis* in the Mediterranean, enhancing our understanding of fine-scale kinship, maternal lineage structure, breeding habits and broader population dynamics.

The use of the 3′ tandem repeat region within the mtDNA D-loop revealed over 40 distinct haplotypes among 139 individuals, despite the overall low diversity previously documented in Mediterranean populations [4,9]. Distinct yet related heteroplasmic patterns were observed among siblings from a single nest (Figure 1b), consistent with the mitochondrial bottleneck effect, whereby multiple mtDNA molecules are unequally transmitted across generations within the maternal lineage [23]. A combination of gel electrophoresis and sequencing confirmed subtle size and sequence variation across clutches. Although no consistent correlation was observed between haplotype structure and geographic origin, the D-loop repeat region provided exceptional resolution for maternal lineage assignment and individual-level discrimination based on heteroplasmic profiles. In our study, it served as a valuable complementary tool for detecting nest-site fidelity and mapping reproductive outputs of individual females.

Single-nucleotide polymorphism (SNP) analysis via double-digest RAD sequencing (3RADseq) substantially improved our capacity to resolve both fine-scale kinship and broader population structure. After stringent quality filtering, over 6000 high-confidence SNPs were retained, providing sufficient resolution to distinguish closely related individuals and delineate population-level differentiation. Pairwise Fst values indicated moderate to high genetic differentiation among the four sampled populations, with the lowest divergence observed between Alexander and Taninim (Fst = 0.084; ΦPT = 0.131), suggesting some degree of gene flow or recent shared ancestry. In contrast, the Turkish population exhibited pronounced genetic separation, consistent with long-term drift and isolation in a distinct biogeographic region. An alternative explanation for this divergence is the possibility of recent demographic bottlenecks. While our data and sampling breadth make this less likely, we cannot exclude it without more extensive temporal and geographic sampling. The Hula samples, originating from an artificially introduced and now declining population, showed markedly elevated differentiation in both Fst and ΦPT values—indicative of a severe bottleneck and founder effect.

PCA results further confirmed distinct clustering of Turkish and Hula samples, with the Israeli populations (Alexander and Taninim) exhibiting partial overlap, likely reflecting historical gene flow or more recent translocation events. Importantly, AMOVA revealed that the majority of genetic variation (68%) is attributable to genetic variability among individuals, with a smaller but significant proportion (18%) explained by differences among populations. This distribution highlights both the high individual-level genetic heterogeneity within populations and the regional genetic structuring that reflects fragmentation across the species’ range.

It is important to note that the Alexander population constitutes the only dataset with a sufficiently large number of individuals to enable reliable statistical analyses of genetic structure and kinship. In contrast, the other populations, and particularly the Turkish cohort (*n* = 8), were represented by very few samples. Such limited sample sizes reduce statistical power and increase the risk that observed differentiation reflects stochastic sampling effects rather than true population-level patterns. Therefore, these smaller groups should be interpreted primarily as outgroup references, providing context for how divergent or similar they are relative to the Alexander population, rather than as fully representative populations. This asymmetry in sampling effort underscores the need for future studies to expand geographic and temporal coverage in order to robustly characterize genetic variation across the species’ range.

Normalized-IBS (Identity by State) matrices enabled precise quantification of genetic similarity among individuals and allowed direct inference of full- and half-sibling relationships. As expected, the Alexander and Taninim populations showed the highest levels of similarity. Heatmaps (Figure S1, Figure 3) identified clusters of full siblings, with shared mtDNA haplotypes corroborating maternal assignment. Eight samples from Türkiye were included to provide a broad comparison with the Israeli populations. Given the large geographic range of Türkiye, these samples should not be interpreted as representing a single cohesive population. The Turkish–Israeli differences observed here should therefore be considered preliminary. The Alexander River population, the focal group of this work, was used to study reproductive and population dynamics. From the Normalized-IBS matrix and mtDNA data, we identified 43 samples involved in 47 full-sibling relationships, averaging a value of 0.49 ± 0. In addition, 11 samples were involved in 13 half-sibling relationships within nests, averaging a value of 0.30 ± 0.These values correlate well with previous kinship analysis in sea turtles, suggesting that the Alexander population exhibits genetic variation typical of a healthy, outbred group, with no clear signs of elevated relatedness or inbreeding.

The combined mtDNA and Normalized-IBS values enabled the detection of at least 45 distinct females within the Alexander River population, 44 of them over a period of 5 consecutive years (Table S1). Next, we tried to detect half-siblings that did not share the same mother. We estimated the involvement of 37 males, 36 of them during this 5-year period, in mating events. Our current findings estimate that about 80 individuals are involved in mating events is already much larger than the Israeli NPA estimated population of 50.

Our sampling method is restricted to dead hatchlings left in hatched nests. Between 2017 and 2021, there were 131 recorded nests in the Alexander River banks. We have sampled only 51 nests. Some of the nests contained one or very few samples, minimizing the probability of detecting more than one father. Since only 38% of the nests were sampled, and not all the population is involved in mating, we expect this number to be higher, especially for the males. These numbers far exceed field-based visual estimates, suggesting that traditional demographic surveys may grossly underestimate effective population size in cryptic aquatic species such as *T. triunguis*. Notably, these estimates also emphasize the continued reproductive activity in what was thought to be a heavily depleted population post−1992 flood [14] and affirm the conservation value of the Alexander population as a potential genetic reservoir.

The kinship data revealed several interesting breeding behaviors. The presence of half-siblings within a single turtle nest provides strong genetic evidence of multiple paternity, a phenomenon increasingly documented across various turtle species. This reproductive strategy, where a female mates with multiple males within a breeding season, is now widely recognized as an important mechanism for maintaining or enhancing genetic diversity, particularly in small or isolated populations [26,27]. Multiple paternity can reduce the risk of inbreeding, increase offspring heterozygosity, and enhance the adaptive potential of populations facing environmental change or demographic bottlenecks [28,29].

We also detected multiple clutches laid by the same female in the same year. Potential mother #26 laid nest 13 on 29 May 2021, nest 37 on 7 June and nest 2 one week later. Potential mother #25 laid two nests—10 days apart. The presence of full siblings from different fathers across temporally separated nests (e.g., nests 14 and 19; 13 and 37) may provide evidence for sperm storage in *T. triunguis*. This reproductive strategy has been widely documented across turtle taxa [30,31] and is well established in closely related softshell species such as *Pelodiscus sinensis* [32,33]. We also find evidence of males involved in multiple mating events, some of them detected over the whole 5-year period.

Multiple paternity is harder to confirm when males are closely related. A PCA of two nests from the Hula Nature Reserve revealed a clear genetic separation between nests, as well as internal sub structuring within Nest 2 (2a and 2b). Normalized-IBS values (0.50 and 0.43 within groups, 0.32 between subgroups) supported multiple paternity. Given the artificial origin of the Hula population and subsequent relocation of hatchlings, this diversity may reflect inbreeding within a very limited founder group.

This study demonstrates that even in genetically constrained populations, reproductive strategies such as multiple paternity, sperm storage, and repeated clutching can maintain considerable fine-scale genetic diversity. These behavioral adaptations may mitigate inbreeding risk at the clutch and nest level. Increasing the sample from other populations will assist in understanding the connectivity between the Israeli populations and the differences from remote populations in the Mediterranean and Africa.

Conservation efforts should aim to preserve both population size and natural reproductive patterns. Ongoing genetic monitoring is vital to detect changes in mating systems, lineage loss, or demographic shifts. If evidence of inbreeding or reduced genetic diversity emerges, managed gene flow may be necessary. Key reproductive indicators, such as multi-paternal clutches and female site fidelity, should be used to assess population health and guide adaptive management.

## 4. Materials and Methods

### 4.1. Sample Collection and DNA Extraction

**Figure 5:**
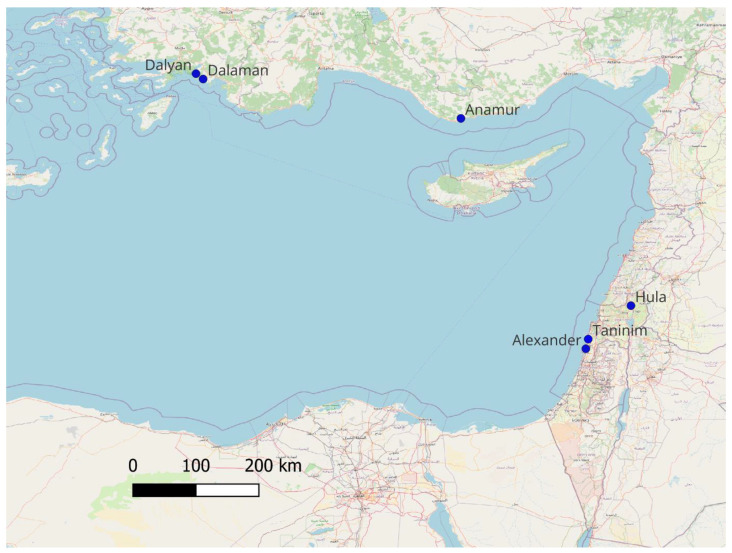
Map of the eastern Mediterranean Basin showing all sampling sites in Türkiye (Anamur River, Dalyan Strait, and Dalaman River) and Israel (Hula Nature Reserve, Taninim River and Alexander River) used in this study.

A total of 139 *T. triunguis* DNA samples were obtained from both dead and living hatchlings. Of these, 89 deceased hatchlings were collected from the Alexander River, 8 from the Taninim River, and 8 from various sites in Türkiye, including two samples each from the Anamur River, Dalyan Strait, and Dalaman River, as well as two of unknown origin. Additionally, 34 samples were collected from live hatchlings emerging from two nests at the Hula Nature Reserve (Figure 5). Small tissue biopsies from the ventral side of the carapace were taken from the hatchlings as part of their veterinary health check prior to their planned relocation from the Hula Nature Reserve to coastal river habitats, in accordance with INPA’s management protocol. Precautions were taken to ensure that any potential harm to individual turtles or to the species as a whole was thoroughly considered and minimized. Because the Turkish dataset was small (*n* = 8) and included two quality control individuals, results involving Türkiye are preliminary and intended for broad comparison only.

Genomic DNA was extracted from all samples using the DNeasy^®^ Blood & Tissue Kit (QIAGEN, Hilden, Germany), following the manufacturer’s protocol. DNA concentration and purity were initially assessed using a NanoDrop™ One/OneC Microvolume UV-Vis Spectrophotometer (Thermo Fisher Scientific, Waltham, MA, USA), and integrity was verified by 1% agarose gel electrophoresis stained with RedSafe (run at 110 V for 45 min). For precise quantification, DNA concentrations were measured using a Qubit 2.0 Fluorometer (Thermo Fisher Scientific, Waltham, MA, USA) with the dsDNA High Sensitivity (HS) Assay and the Quant-iT™ PicoGreen™ dsDNA Assay Kit (Thermo Fisher Scientific, Waltham, MA, USA). All samples were normalized to a final concentration of 20 ng/μL for downstream applications.

### 4.2. mtDNA Haplotyping

A variable repeat region (“TATATATC”) located at the 3′ end of the mitochondrial genome was PCR-amplified using flanking primers TT-D-864F (5′-TTGCGCAGTCCAATACATACA-3′) and TT-D-1110R (5′-CAGTGCCATGCTTTGTGTTAAG-3′). PCR reactions (20 μL) contained 1× DreamTaq PCR Master Mix (Thermo Fisher Scientific, Waltham, MA, USA), 0.5 μM of each primer, and ~40 ng of template DNA. Amplified fragments (193–297 bp; 13–26 copies of the 8-bp repeat motif) were visualized on 1.5% agarose gels. A subset of amplicons exhibiting distinct size polymorphisms was selected for bidirectional Sanger sequencing on an ABI 3730 DNA Analyzer (Applied Biosystems™, Foster City, CA, USA). Forward and reverse sequences were edited, aligned, and compared using BIOEDIT v7.2.5 [34]. The determination of mtDNA haplotypes was therefore based on repeat number estimated by both gel electrophoresis and sequencing.

### 4.3. RAD Laboratory

The 3RAD libraries were prepared following the protocol described by Hoffberg et al. [35]. Briefly, 10 µL of genomic DNA per sample was digested with the restriction enzymes XbaI and EcoRI-HF, along with a third adapter-dimer cutting enzyme, NheI-HF (New England Biolabs, Ipswich, MA, USA), at 37 °C for 2 h. Adapters specific to each restriction site (XbaI and EcoRI) with unique inline barcodes were ligated immediately after digestion. T4 DNA ligase was added directly to the digestion mix, allowing simultaneous ligation and digestion of adapter dimers and chimeric products during temperature cycling. Post-ligation, libraries were pooled based on DNA quality and purified using 1.8× CleanPCR magnetic beads (CleanNA, Waddinxveen, The Netherlands). DNA concentration was quantified using the Qubit 2.0 fluorometer and the dsDNA HS Assay Kit (Thermo Fisher Scientific, Waltham, MA, USA). Fragment size selection (310–540 bp) was performed with a BluePippin system using a 1.5% cassette and R2 marker (Sage Science, Beverly, MA, USA), based on in silico digestion of the *Pelodiscus sinensis* genome. This size range was predicted to yield approximately 20,426 loci when using the selected enzymes [36]. A single-cycle PCR with the iTru5_8N primer was performed to complete adapter sequences and incorporate unique molecular identifiers. This was followed by a limited-cycle indexing PCR using a P5 short primer and a P7 indexing primer to finalize the library. Final library concentrations were quantified with Qubit 2.0, and fragment size distributions were assessed using the Agilent 2100 Bioanalyzer with the High Sensitivity DNA kit (Agilent Technologies, Santa Clara, CA, USA). Sequencing was conducted using the Illumina NovaSeq SP platform (2 × 150 bp) (Illumina, San Diego, CA, USA), targeting a minimum of 30× coverage per individual, at the MDC Genomics Center (Berlin, Germany).

### 4.4. RAD Analysis

Illumina reads were initially demultiplexed using the P7 indexes, and the quality of the raw sequence data was assessed using FastQC 0.12.0 [37]. Adapter trimming was then performed with Cutadapt 5.1 [38]. Reads pooled with inline P5 and P7 barcodes were further demultiplexed using Flexbar 3.5 [39]. PCR duplicates were removed using the Python 2.7 script Filter_PCR_duplicates.py. Paired-end reads were then merged using PEAR [40] with the parameters −v 30 (minimum overlap size), −n 20 (minimum read length), and −m 190 (maximum read length), to filter out merged reads longer than the target range for R2SCO loci (213–309 bp). Unassembled reads were subsequently quality-filtered (Q > 30) and trimmed to a maximum length of 130 bp using Trimmomatic 0.32 [41], based on FastQC results. The custom script Check_Restriction_Site.py verified the expected restriction sites, CTAGA (XbaI) and AATTC (EcoRI), at the read pair ends. A final filtering step was performed using Filter_Reads.py, which removed read pairs containing internal, undigested restriction sites (XbaI, EcoRI, or NheI), thus excluding incompletely digested or chimeric fragments. The number of reads retained at each processing step is summarized in Table S2.

### 4.5. Reference Construction and SNP Calling

3RAD sequences were analyzed using a reference dataset, based on three *T. triunguis* individuals. Two unrelated individuals from the Alexander River—representing the largest known Israeli population—and one outgroup individual from Türkiye. These individuals were sequenced with MiSeq 300 bp paired-end reads for reference generation. The reference was created following the protocol described by Driller et al. [25], producing a high-quality set of reduced-representation single-copy orthologs (R2SCOs). This pipeline was designed to generate references for non-model organisms lacking a reference genome, enabling the application of the Stacks reference-based pipeline.

The resulting reference contained 20,426 R2SCO loci ranging in size from 213 to 309 bp (Figure S3). Following read preprocessing and quality control, the cleaned reads were mapped to the R2SCO-XbaI-EcoRI-213–309 reference using Bowtie2 v2.3.4.1 [41]. Mapping was performed with default parameters, along with the flags –no-mixed and –no-discordant to ensure that only properly paired reads aligning to consistent genomic positions were retained in the resulting SAM files. The –no-mixed flag excluded read pairs where only one mate aligned, while –no-discordant excluded improperly oriented or spaced pairs, thus filtering for reliable alignments.

Mapped reads from 140 samples (from 139 individuals) with sufficient alignment rates and mean coverage above ~10× were processed using the reference-based pipeline ref_map.pl from Stacks v2.53 [42]. Variant detection and genotype calling were performed using gstacks, which identified SNPs and assigned genotypes for each individual. The populations module was subsequently employed to calculate population genetic parameters. A locus was retained only if it was genotyped in at least 60% of individuals within a population (-r 0.6). The parameter (-p 1) was used to require loci to be present in at least one population for inclusion.

In total, two VCF files were generated and analyzed: (1) the full dataset (*n* = 140), including a duplicated individual and a dataset including only Hula individuals (*n* = 34). These outputs included SNPs, haplotypes, and various population genetic statistics for downstream analyses. Since the duplicated individual analysis showed almost identical results (as shown in Figure 2), only one of the two was used for analysis.

### 4.6. SNP Filtering and Population Genetic Analyses

To evaluate appropriate filtering thresholds, various summary statistics were calculated from each VCF file using VCFtools [43], including site and individual depth (–depth, –site-mean-depth), missingness (–missing-site, –missing-indv), allele frequencies (–freq2), and heterozygosity (–het). These were inspected in RStudio v2023.06.0 using the tidyverse package [44].

VCFs were filtered in VCFtools with the following criteria:

Full dataset: min. mean site depth 10×, max. mean site depth 130×, genotype depth 10–130×, MAF ≥ 0.02, max. missingness 10%.

Hula-only dataset: min. mean site depth 10×, max. mean site depth 190×, genotype depth 10–190×, MAF ≥ 0.05, max. missingness 5%. The number of high-quality SNPs retained after filtering was 6115 for the full dataset and 3565 for the Hula-only dataset.

An Analysis of Molecular Variance (AMOVA) was performed in GenAlEx [45] to partition genetic variation between populations based on sampling locations [46,47]. Wright’s Fst [48] was calculated using 999 permutations for significance testing.

Principal Component Analysis (PCA) was performed using PLINK 1.9 (–pca) [49,50] after linkage disequilibrium (LD) pruning with the command –indep-pairwise 50 5 0.2, which removed SNPs with pairwise R^2^ > 0.2 within a 50-SNP window sliding by 5 SNPs. The number of SNPs retained post-pruning was 1791/6115 and 1046/3565 for the two datasets, respectively.

### 4.7. Kinship Analysis

Genetic similarity between individuals was assessed using a comprehensive analysis of all available SNPs. Similarity values were assigned using a custom Python script (main.py) developed by Ohana et al. [20], which scored genotypes as follows: 1 when two individuals shared the same genotype, 0.5 when they shared one allele (i.e., heterozygous match), and 0 when they had completely different alleles (opposite homozygotes). Identity-by-state (IBS) values were normalized to range from 0 (unrelated) to 1 (identical). We applied the normalization method described by Ohana et al. [20], where a value of 1 represents identical samples and 0 corresponds to the average IBS value observed between the two most genetically distinct nests or families in the dataset. Normalized-IBS values were calculated using the formula: Normalized IBS = (IBS value − av. min)/(1 − av. min) where av. min is the average IBS value between all individuals from the two most dissimilar nests.

## 5. Conclusions

The integration of mitochondrial tandem repeat-region profiling with genome-wide SNP analysis has yielded unprecedented insight into the breeding biology, kin structure, and population dynamics of *T. triunguis*. Our findings reveal a complex pattern of reproductive behavior and highlight the genetic resilience of this threatened species, even in fragmented or anthropogenically impacted habitats. The ability to accurately estimate effective population size, genetic diversity, and levels of inbreeding provides critical tools for monitoring conservation status. These results emphasize the importance of genomically informed and behaviorally grounded conservation strategies to safeguard the long-term viability of *T. triunguis* in the Mediterranean.

## Figures and Tables

**Table 1:** Matrix of pairwise Fst (below diagonal) and ΦPT (above diagonal) based on allelic and genotypic data from filtered SNPs for four populations based on geographic sampling locations of *T. triunguis*.

	Alexander (*n* = 89)	Taninim (*n* = 8)	Hula (*n* = 34)	Türkiye (*n* = 8)
Alexander		0.131	0.288	0.251
Tanninim	0.084		0.395	0.202
Hula	0.187	0.242		0.462
Türkiye	0.170	0.144	0.302	

Note: Fst values below diagonal, ΦPT values above diagonal, *p* value (*p* < 0.001). *n* = sample size per population.

**Table 2:** Matrix of pairwise average Normalized-IBS (Identity by State) between geographic sampling locations of *T. triunguis*.

	Alexander	Taninim	Hula
Tanninim	0.12		
Hula	0.06	0.04	
Türkiye	0.00	−0.01	−0.04

*n* = sample size per population: Alexander = 89, Taninim = 8, Hula = 34, Türkiye = 8.

## Data Availability

All data have been made available on the NCBI Sequence Read Archive (SRA) database under BioProject PRJNAAll scripts used in the 3RAD analysis are available at https://github.com/Gilitiko/RAD-Seq-Scripts. Other relevant data (e.g., VCF files, Supporting Information) are available on Figshare: https://figshare.com/articles/dataset/The_Mediterranean_habitat_of_the_Nile_soft-shelled_turtle_i_Trionyx_triunguis_i_/29930822.

## Supplementary Materials

The supporting information can be downloaded at: https://www.mdpi.com/article/10.3390/ijms26188822/s1.

## Abbreviations

The following abbreviations are used in this manuscript:

NPA	Nature and Parks Authority
IBS	Identity by State
mtDNA	Mitochondrial DNA
